# The New York Observer

**Published:** 1876-12

**Authors:** 


The News without Poison.—The *New York Observer* claims to publish the *best family newspaper*, and repudiates all unsound or objectionable teaching. Even its advertising columns are free from all quackery and dangerous teaching, and the whole paper is filled with pure and entertaining reading. The price, $3.15 a year post paid, will be found a good investment. S. I. Prime & Co., 37 Park Row, New York.

